# Risk Factors and Complications of Childhood Obesity and Overweight in an Urban Setting of a Lower Middle-Income Country

**DOI:** 10.3390/ijerph22111697

**Published:** 2025-11-10

**Authors:** Varun Govind Krishna, Sarala Rajajee, Venkatakrishna Rajajee, Hemchand K. Prasad

**Affiliations:** 1 Lyman Briggs College, Michigan State University, East Lansing, MI 48825, USA; krish150@msu.edu; 2 Department of Pediatrics, Dr. Mehta’s Hospitals, Chennai 600031, India; saralarajajee@yahoo.com; 3 Center for Global Health, University of Michigan, Ann Arbor, MI 48109, USA; 4 Division of Pediatric Endocrinology, Department of Pediatrics, Dr. Mehta’s Hospitals, Chennai 600031, India; hemchan82@gmail.com

**Keywords:** childhood obesity, childhood overweight, risk factors, cardiovascular risk factors, developing nations

## Abstract

In contrast to several high-income nations, childhood obesity prevalence is rising in low/middle-income countries. Our objective was to study risk factors and complications of childhood overweight/obesity in an urban lower middle-income country setting. This was an observational study. Children aged 2–18 years at a pediatric clinic in Chennai, India were enrolled over a 12-month period. The definition of overweight was >23rd and obesity >27th adult equivalent percentile Body Mass Index. Parents and children completed a risk-factor questionnaire. Children with obesity/overweight were evaluated for complications. Of 103 children enrolled, 61% were obese/overweight and 39% healthy weight. Independent predictors of absence of overweight/obesity were as follows: never/rarely consuming sugar-sweetened beverages, never/rarely eating out, and sleep duration > 11 h. Exercise performed rarely/never independently predicted overweight/obesity. No significant difference was observed with screen time or a vegetarian diet. Complications in 54 obese/overweight children included prediabetes (15%), hypertension (11%), dyslipidemia (22%), nonalcoholic fatty liver disease (22%), acanthosis nigricans (24%), and anxiety/depression (17%). In conclusion, differences were observed in behaviors associated with childhood obesity in an urban lower middle-income environment compared to those in high-income nations. Behaviors associated with childhood obesity in an urban lower middle-income environment are similar to those reported from high-income nations, with some differences. Complications of overweight/obesity are common in this setting.

## 1. Introduction

Childhood obesity has major health implications, including an increased risk of death in adulthood, mostly from cardiovascular disease [1]. Globally, the prevalence of obesity has increased [2]. Traditionally, childhood obesity has predominantly been a problem of high-income nations such as the United States [3], while the major nutritional concern in low- and middle-income countries (LMICs) has been under-nutrition. In recent years, however, these trends have reversed. The prevalence of childhood obesity in some high-income nations may have reached a plateau [4,5,6,7,8]. Meanwhile, despite the persistence of under-nutrition as a major problem, several LMICs, such as India, have seen a rate of increase in childhood obesity 30% higher than in high-income nations [9]. Public health efforts to limit the long-term impact of childhood obesity worldwide should therefore refocus on LMICs.

The prevalence of childhood obesity in India is estimated at 8%, and the prevalence of childhood overweight at 12% [10]. Several modifiable risk factors for childhood overweight and obesity have been identified, primarily in studies from high-income nations [11,12,13,14,15,16,17,18,19,20,21], but also in some studies from LMICs [22,23,24,25,26]. Studies in high-income nations have suggested a link with consumption of sugar-sweetened beverages [11,12,13], television watching [14,15,16], other forms of screen time such as video games [15,17], insufficient sleep [18,19,20,21], and later bedtime [19]. In addition to risk factors studied primarily in high-income settings, descriptive studies from LMIC settings suggest risk factors such as energy-dense high-calorie foods [22,23,24,27,28], a non-vegetarian diet [22], and limited physical activity [22,23,24,25,26,27] are common in children with overweight and obesity. However, the contribution of these risk factors to childhood obesity and overweight in LMIC settings has not been established using analytical studies. In contrast to high-income nations such as the United States, where children in rural regions are at higher risk of obesity [29], the risk of obesity in LMICs may be higher in urban regions because of the greater availability of sugar-sweetened beverages and high-calorie processed food [30]. Recent studies have demonstrated an increased risk of obesity among adults who migrated from rural to urban regions in countries such as Peru and India [31,32]. The prevalence of childhood obesity in urban regions in India was estimated at 9% in a 2024 systematic review, compared to 4% in rural regions [33]. The prevalence of major complications of childhood obesity has also primarily been established in high-income nations. These include hypertension [34,35,36], dyslipidemia [34,35,36,37], type 2 diabetes mellitus [34,36,38], metabolic syndrome [39], cardiovascular disease [40], and nonalcoholic fatty liver disease (NAFLD) [41]. There is limited corresponding data on the consequences of obesity in LMICs, although similar complications have been described in smaller studies [42,43].

Knowledge of risk factors and consequences of childhood obesity in LMICs is essential to prioritize high-impact prevention efforts. Our primary objective, therefore, was to prospectively evaluate risk factors among children with overweight and obesity in an urban LMIC setting. Our secondary objective was to establish the burden of common complications of childhood obesity and overweight in this setting.

## 2. Materials and Methods

Approval of the Dr. Mehta’s Hospital institutional review board (IRB/MCH/24/2018) was obtained for this observational study. This study complies with the World Medical Association Declaration of Helsinki regarding ethical conduct of research involving human subjects and/or animals. Written informed consent from parents and the assent of children, where appropriate, was obtained. This was an observational study to determine risk factors for obesity/overweight and document the prevalence of complications of obesity/overweight. The setting was an urban referral center in Chennai, India, a lower middle-income country. Subjects were consecutive children between the ages of 2 and 18 years evaluated at a pediatric referral clinic during a 12-month period, July 2019–June 2020. Sample size was estimated using preliminary data from the clinic on expected patient volume, prevalence of obesity/overweight in this referral population, and prevalence of risk factors [44]. Assuming 2 obese/overweight children per healthy weight child in this tertiary referral population, 80% power (1 − β = 0.8) at the 5% level of significance (α = 0.05) and presence of the risk factor in 30% of the healthy weight group, the sample size to detect an Odds Ratio (OR) of 3.5 was estimated at 95. Study subjects were screened and enrolled during referral visits specifically for the management of overweight or obesity, and during well-child visits of otherwise healthy children. In accordance with Indian Academy of Pediatrics (IAP) guidelines, overweight was defined as the 23rd and obesity as the 27th adult equivalent percentile Body Mass Index (BMI) [45]. Overweight and obesity in children < 5 years of age were defined as a weight-for-height greater than 2 and 3 standard deviations above the World Health Organization (WHO) Child Growth Standards median, respectively [46]. Exclusion criteria included (1) a primary endocrine disorder that may cause obesity, including but not limited to cortisol excess (Cushing’s syndrome), hypothyroidism, growth hormone deficiency, pseudohypoparathyroidism type 1a, and hypothalamic cause obesity; (2) prolonged (>1 month) use of medications that may result in obesity, such as psychoactive drugs (such as olanzapine, risperidone), antiepileptic drugs (such valproate), and glucocorticoids; (3) established diagnosis of type 1 diabetes or familial dyslipidemia and (4) intracranial tumors. Overweight and obesity were additionally assessed in all subjects using Centers for Disease Control and Prevention (CDC, United States) [47], WHO [46,48], and International Obesity Task Force (IOTF) criteria [49]. The frequency of overweight and obesity among patients included in our study was not expected to reflect community prevalence since children were frequently referred to the clinic for the management of obesity.

Subjects were provided a questionnaire (Supplementary S1) with 14 questions in 4 sections related to diet, physical activity, screen time, and sleep. In the absence of validated instruments to evaluate pediatric obesity risk factors in an urban Indian environment, the questionnaire was developed ad hoc for this setting. The questionnaire was completed in the clinic waiting room primarily by parents, but with the involvement of the child. The 7 diet-related questions addressed consumption of a non-vegetarian diet, sugar-sweetened beverages, meals prepared outside the home, fried foods, rice as a major part of the meal, servings of vegetables, and servings of fruit. All meals cooked and purchased outside the home, including meals at restaurants, and meals purchased outside but consumed at home, were included in the “eating out” definition. School lunches were excluded from this definition. The single question on exercise addressed the frequency (per week) of physical activity sufficient to make the child breathe hard for at least 20 min. The 4 questions related to screen time related to daily hours of television, computer, mobile phone/tablet, and video-game screen exposure. The 2 questions related to sleep addressed bedtime and hours of sleep. All children were screened for hypertension in accordance with recent guidelines [50]. The term “Never/very rarely” referred to behaviors that occurred no more frequently than once a month. All children with obesity and overweight were evaluated by a pediatric endocrinologist (H.K.P.). Laboratory evaluation and ultrasound of the abdomen were recommended for children with obesity and overweight. Laboratory evaluation included a fasting glucose, fasting lipid profile, hemoglobin A1C, electrolytes, renal function, hepatic function, and hormone levels. A clinical evaluation was performed for the following complications of obesity, with solicitation of symptoms and a physical examination only: obstructive sleep apnea (OSA), NAFLD, acanthosis nigricans, slipped capital femoral epiphysis (SCFE), tibia vara, polycystic ovarian syndrome (PCOS), hyperandrogenism (in females), anxiety, depression, and any other condition considered appropriate. The criteria used for the diagnosis of these conditions, where appropriate, are in Table 1. NAFLD was diagnosed based on an ultrasound image demonstrating fatty infiltration, with no alternate etiology, and no clinical evidence of cirrhosis. Biopsy was not routinely performed. The diagnosis of other complications was made using standard criteria.

Statistical analysis: Descriptive statistics included proportions for categorical variables and median with interquartile range (IQR) for continuous variables. Associations between variables and outcome (overweight/obesity) were tested for statistical significance using the chi-square or Fisher exact test for categorical variables and the Mann–Whitney U-test for continuous variables. Multivariate analysis was performed using logistic regression, with the presence of overweight or obesity as the response variable. Explanatory variables included age, sex, and risk factors evaluated in the survey, selected based on prior studies and biological plausibility. An Odds Ratio (OR) with 95% confidence interval (95% CI) was calculated for variables that attained statistical significance in multivariate analysis. A complete case analysis approach was used for missing data. Subjects with missing values for any of the variables used in a specific analysis were excluded from that calculation. The threshold for statistical significance was a two-sided *p* < 0.05. Statistical analyses were performed using MedCalc Statistical Software version 19.2.6 (MedCalc Software bv, Ostend, Belgium).

## 3. Results

A total of 103 children met eligibility criteria for the study and were enrolled; of these, 63 (61%) met IAP criteria for obesity/overweight and 40 (39%) for healthy weight. A further four children were excluded due to the presence of hypothyroidism. Five cases were excluded from the final analysis of predictors of obesity/overweight because of missing data. The prevalence of overweight and obesity using CDC, WHO, and IOTF criteria is in Table 2.

Among children with obesity/overweight, 48 (76%) met IAP criteria for obesity and 15 (24%) for overweight. The distribution of baseline variables is in Table 3.

### 3.1. Risk Factors

The distribution of responses to questions about risk factors in obese/overweight and healthy-weight children is in Table 4.

Responses to questions regarding the following risk factors demonstrated no statistically significant difference between children with obesity/overweight and healthy weight (Table 4): non-vegetarian diet, consumption of rice, vegetable consumption, fruit consumption, television watching, computer use, use of mobile phones, hours playing video games, and bedtime. A statistically significant difference was present in responses to questions regarding the following risk factors between children with overweight/obese and healthy weight: consumption of sugar-sweetened beverages, eating out, consumption of fried food, physical activity, and hours of sleep. Results of multivariate analysis, including these variables, age, and sex, are in Table 5, using various international criteria for overweight/obesity. Eating meals prepared outside the home 1–2 times per week was an independent predictor of obesity/overweight regardless of diagnostic criteria. The consumption of sugar-sweetened beverages 5–7 times per week was an independent predictor with all diagnostic criteria except those of WHO. Physical activity five or more times a week and sleep duration of 11 h or more both had a protective effect against obesity/overweight, regardless of diagnostic criteria. Overall, in this setting, eating meals prepared outside the home emerged as the strongest risk factor for obesity/overweight, while physical activity and adequate sleep duration had the strongest protective effect.

### 3.2. Complications

**Figure 1:**
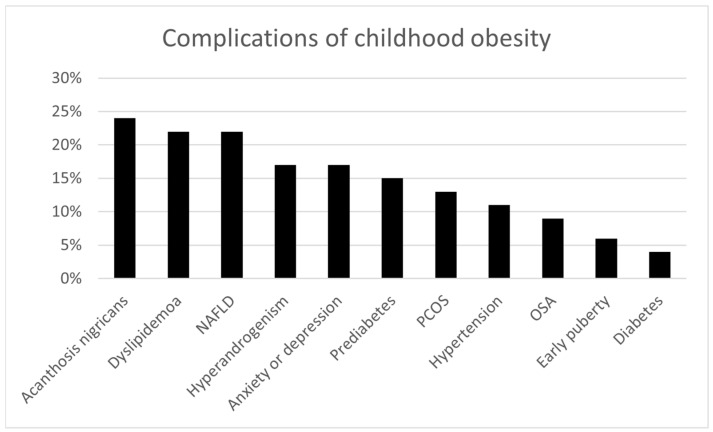
Complications of childhood obesity present among 54 children with obesity/overweight (24 male, 30 female) who underwent systematic evaluation. NAFLD = nonalcoholic fatty liver disease. PCOS = polycystic ovarian syndrome. OSA = obstructive sleep apnea.

The following complications were present among 54 children with obesity/overweight (24 male, 30 female) who underwent systematic evaluation (Figure 1)—prediabetes in 8 (15%), diabetes in 2 (4%), hypertension in 6 (11%), dyslipidemia in 12 (22%), OSA in 5 (9%), NAFLD in 12 (22%), acanthosis nigricans in 13 (24%), PCOS in 4 (13% of females), hyperandrogenism in 5 (17% of females), early puberty in 3 (6%, 1 male and 2 females), and anxiety or depression in 9 (17%). Among the 12 children with dyslipidemia, the following abnormalities were seen: 9 (75%) with elevated triglycerides, 6 (50%) with low high-density lipoprotein, 5 (42%) with high cholesterol, and 5 (42%) with elevated low-density lipoprotein.

## 4. Discussion

Obesity in LMIC nations is an increasingly important global public health concern. It is therefore critical to identify the highest-yield targets for prevention efforts in these settings. It is also important to establish that the health consequences of obesity in LMICs are comparable to those observed in high-income nations. Consistent with studies from high-income nations [11,12,13], our study identified the consumption of sugar-sweetened beverages as an independent risk factor. Of note, we identified eating out as a specific, independent risk factor in this urban Indian environment. Physical inactivity was also independently predictive of overweight/obesity. Similar to studies from high-income nations [2,18,21], increased sleep duration was beneficial; however, in contrast to some studies from high-income settings [19], bedtime was not. In contrast to some studies from high-income nations, recreational screen media time was not associated with childhood obesity in this urban Indian setting. Up to 39% of the Indian population may be vegetarian [51], and some studies have suggested a vegetarian diet may be associated with a lower risk of pediatric obesity in this setting [22]. However, this association was not present in our study. Our study also did not identify a statistically significant association between overweight/obesity and rice consumption, vegetable consumption, or fruit consumption. The most common health consequences of overweight/obesity were dyslipidemia, NAFLD, acanthosis nigricans, prediabetes, hypertension, and hyperandrogenism in females. Our findings are consistent with those of other studies from India, as well as other LMICs [10,23,24,26,27,28,30,33,42,52,53,54,55,56,57,58]. These studies have also highlighted dietary changes, high-calorie foods, lack of physical activity, and insufficient sleep as risk factors for childhood obesity. Strengths of our study relative to others in similar settings are its prospective analytical design, inclusion of healthy-weight children to permit comparison, and adjustment for confounders in multivariate analysis. In contrast to other studies, our focus was on behaviors and modifiable risk factors.

Rapidly growing economies such as India have seen a large expansion in the middle-class, and therefore the capacity to purchase and consume food prepared and packaged outside the home, rather than traditional Indian home-cooked meals [52,59]. A 2017 survey of 13,274 schoolchildren in the age group 9–17 years conducted by the Centre for Science and Environment in urban areas across India revealed that 49% of children consumed packaged sugar-sweetened beverages more often than twice a week, and over half consumed sweet or salty packaged food more than twice per week [59]. Our study suggests a link between these changes in food consumption and the rising epidemic of childhood obesity. Eating meals prepared outside the home (excluding school lunches) was an independent predictor of childhood overweight/obesity in our study. It is likely that “eating-out” is a surrogate for greater consumption of packaged and ultra-processed food high in fat (especially saturated and trans-fat), sugar, and salt (HFSS), while lacking in micronutrients and minerals [52]. Processed foods cause obesity because they are designed to be energy-dense, with taste-enhancing ingredients that override signals of fullness and cause overconsumption [60]. Sugar-sweetened beverages likely lead to obesity by providing a large amount of calories from rapidly absorbed sugars, without providing satiety [61,62]. As a result, calorie intake from solid food is not reduced during meal-time to compensate for the liquid calories.

Our finding that physical inactivity increased the risk of overweight/obesity is consistent with other studies from India [22,23,24,25,26], as well as higher-income nations [63,64,65,66]. Physical activity likely decreases obesity by burning calories, boosting metabolic health, and increasing muscle mass [63]. Recommendations for increased physical activity for the prevention of childhood obesity include mandatory physical education at school, structured play for younger children, and participation in organized sports for school-age children [63]. Our finding that increased sleep duration had a protective effect against childhood obesity/overweight is consistent with studies from high-income nations [67,68], as well as more recent data from LMICs [57]. Adequate sleep and restoration of the circadian rhythm likely decrease the risk of obesity through appetite hormone regulation, decreasing eating opportunities at night, and preventing daytime fatigue that may impact physical activity [68]. Efforts to optimize sleep duration may focus on promoting independent sleep, consistent sleep routines, and avoiding caffeine or sugary drinks [69]. At a policy level, later school start times will improve sleep duration [70].

All these risk factors—consumption of sugar-sweetened beverages, eating meals prepared outside the home, physical activity, and sleep duration—may be modifiable through education, while some may be addressed through regulation. Recent IAP guidelines were directed toward children and parents, as well as schools and regulatory authorities. The recommendations within these guidelines that address the specific risk factors identified in our study may have the greatest impact if implemented. These include recommendations for consumption of food cooked at home rather than restaurants, avoidance of packaged foods, restricting sugar, and exclusion of trans-fats [52]. Recommendations to schools and regulatory authorities that may have the greatest impact include the exclusion of HFSS foods from school cafeterias, traffic light coding of packaged food, restrictions on advertising of unhealthy food, and tax benefits for healthier food choices [52]. The absence of an association between recreational screen media time and overweight/obesity in our study may be a consequence of under-reporting of screen time by children and parents, or variations in associated behaviors and risk factors compared to high-income nations that may impact the link to childhood obesity. This includes possible differences in physical activity in addition to screen time, eating during screen time, and television advertising of unhealthy food. It should be noted, however, that while several studies from high-income nations have suggested a link between screen time and childhood obesity [14,15,16,17], others from the same settings suggest the association may be modest or negligible compared to better-established risk factors [71,72,73]. While the limitation of screen time in children may be otherwise desirable, the impact of such interventions on childhood obesity is uncertain. No reduction in childhood obesity was seen with the use of interventions to reduce screen time in a recent systematic review [74].

The prevalence of hypertension among children with overweight/obesity in our study (11%) is comparable to the prevalence reported in high-income nations with daytime screening (9%) [35,75]. Of note, studies from high-income settings with the use of ambulatory blood pressure monitoring report hypertension in approximately half of all children with obesity [75,76]. The prevalence of prediabetes (15%) and dyslipidemia (22%) in our study is also comparable to the prevalence reported among children with obesity in the United States [3,36,37]. The high prevalence of cardiovascular risk factors in these children may result in a higher burden of cardiovascular disease in young adulthood and middle-age [77].

Our study has several limitations. The sample size was relatively small. Wide confidence intervals in our study reflect the relatively small sample size. Subjects in our study were enrolled during referral visits for overweight/obesity and well-child clinic visits at a single urban outpatient clinic, and may not be optimally representative of the general population. We did not obtain socio-economic data, such as family income, from our subjects. Our findings cannot be extrapolated to rural settings, where median income may be significantly lower and risk factors quite different. Screening for health consequences of obesity was limited to the conditions described, based on clinical practice at the hospital. Hepatic steatosis was assessed solely by ultrasonography, which limits diagnostic precision.

## 5. Conclusions

Behaviors associated with childhood obesity in an urban lower middle-income environment are similar to those reported from high-income nations, with some differences. Complications of overweight/obesity are common in this setting. Prevention efforts should focus on both the regulation of processed food and sugar-sweetened beverages, as well as efforts directed at increasing physical activity and healthy sleep habits.

## Figures and Tables

**Table 1:** Criteria for diagnosis of complications of childhood obesity.

Complication of Obesity	Criterion
Hypertension	≥95th percentile of systolic or diastolic blood pressure
High total cholesterol	≥200 mg/dL
High Low-Density Lipoprotein (LDL) cholesterol	≥130 mg/dL
Low High-Density Lipoprotein (HDL) cholesterol	<35 mg/dL
High triglycerides	≥150 mg/dL
Prediabetes	Glycated hemoglobin > 5.7%
Diabetes	Glycated hemoglobin > 6.4%

**Table 2:** Frequency of overweight and obesity among 103 children enrolled in the study using various criteria.

Criteria	Obesity	Overweight	Not Overweight/Obese
Indian Academy of Pediatrics (IAP)	48 (47%)	15 (14%)	40 (39%)
World Health Organization (WHO)	39 (38%)	21 (20%)	43 (42%)
Centers for Disease Control and Prevention (CDC)	39 (38%)	15 (14%)	49 (48%)
International Obesity Task Force (IOTF)	29 (28%)	24 (23%)	50 (49%)

**Table 3:** Baseline characteristics of children in the study. IQR—Interquartile Range. BMI = Body Mass Index. BMI percentile for age was calculated using Centers for Disease Control and Prevention (CDC) criteria (United States).

Variable	All Subjects	Obese and Overweight N = 63	Healthy Weight N = 40	*p* Value
Age in years, median (IQR)	8 (5–12)	8.5 (6–13)	7 (5–10)	0.10
Female sex (%)	50 (49%)	32 (51%)	18 (45%)	0.57
BMI in kg/m^2^, median (IQR)	19 (15–24)	23 (20–27)	15 (14–16)	<0.0001
BMI percentile for age by CDC criteria, median (IQR)	91 (42–98)	96 (92–99)	18 (8–46)	<0.0001

**Table 4:** Survey responses among obese/overweight and healthy-weight respondents. *—indicates *p* < 0.05.

Question	Obese/Overweight (%)	Healthy Weight (%)	*p* Value
DIET			
Vegetarian diet	49 (80%)	34 (85%)	0.55
How many times per week the child eats meals prepared outside the home (not including school lunches)			0.002 *
Never/Rarely	15 (25%)	24 (63%)	
1–2	26 (43%)	6 (16%)	
3–4	10 (17%)	5 (13%)	
5 or more	9 (15%)	3 (8%)	
Sugar-sweetened beverages per week			0.02 *
Never/Rarely	27 (45%)	30 (77%)	
1–4	17 (28%)	5 (13%)	
5–7	15 (25%)	4 (10%)	
8 or more	1 (2%)	0 (0%)	
Fried food, number of times per week			0.03 *
Never/Rarely	12 (20%)	19 (48%)	
1–2	24 (40%)	13 (33%)	
3–4	14 (23%)	4 (10%)	
5 or more	10 (17%)	4 (10%)	
Rice as major part of the meal, number of times per week			0.35
Never/Rarely	1 (2%)	0 (0%)	
1–2	1 (2%)	1 (3%)	
3–4	6 (10%)	1 (3%)	
5 or more	18 (30%)	8 (21%)	
Once per day, every day	10 (17%)	12 (31%)	
Twice or more often per day, every day	24 (40%)	17 (44%)	
Vegetable servings, per week			0.58
Never/Rarely	15 (25%)	5 (13%)	
1–2	7 (12%)	7 (18%)	
3–4	11 (18%)	5 (13%)	
5 or more	11 (18%)	10 (25%)	
Once per day, every day	14 (23%)	10 (25%)	
Twice or more often per day, every day	3 (5%)	3 (8%)	
Fruit servings, per week			0.57
Never/Rarely	8 (13%)	7 (18%)	
1–2	9 (15%)	4 (10%)	
3–4	18 (30%)	6 (15%)	
5 or more	13 (21%)	11 (28%)	
Once per day, every day	11 (18%)	10 (26%)	
Twice or more often per day, every day	2 (3%)	1 (3%)	
EXERCISE			
Exercise or play enough to breathe hard for 20 or more minutes per week			0.0009 *
Never/Rarely	32 (53%)	6 (16%)	
1–2	15 (25%)	8 (22%)	
3–4	6 (10%)	9 (24%)	
5 or more	8 (13%)	14 (38%)	
SCREEN TIME			
Screen time—number of hours per school day: Television			0.46
Never/Rarely	9 (15%)	3 (8%)	
<1	13 (22%)	10 (25%)	
1–2	16 (27%)	14 (35%)	
3–4	14 (23%)	11 (28%)	
5 or more	8 (13%)	2 (5%)	
Screen time—number of hours per school day: Computer other than video games			0.13
Never/Rarely	39 (67%)	29 (81%)	
<1	9 (16%)	3 (8%)	
1–2	6 (10%)	1 (3%)	
3–4	0 (0%)	2 (6%)	
5 or more	4 (7%)	1 (3%)	
Screen time—number of hours per school day: Mobile phone or tablet			0.79
Never/Rarely	14 (23%)	11 (28%)	
<1	12 (20%)	8 (21%)	
1–2	19 (31%)	14 (36%)	
3–4	8 (13%)	3 (8%)	
5 or more	8 (13%)	3 (8%)	
Screen time—number of hours per school day: Video games			0.52
Never/Rarely	51 (85%)	34 (90%)	
<1	5 (8%)	2 (5%)	
1–2	2 (3%)	1 (3%)	
3–4	0 (0%)	1 (3%)	
5 or more	2 (3%)	0 (0%)	
SLEEP			
Bedtime			0.30
Earlier than 7:00 PM	2 (3%)	0 (0%)	
Close to 7:00 PM	0 (0%)	0 (0%)	
Close to 8:00 PM	5 (8%)	2 (5%)	
Close to 9:00 PM	10 (17%)	11 (28%)	
Close to 10:00 PM	16 (27%)	15 (39%)	
Close to 11:00 PM	15 (25%)	7 (18%)	
After 11:00 PM	12 (20%)	4 (10%)	
Hours of sleep per night			0.04 *
<4	0 (0%)	0 (0%)	
4–6	8 (14%)	1 (3%)	
6–8	23 (39%)	15 (39%)	
8–10	26 (44%)	16 (41%)	
≥11	2 (3%)	7 (18%)	

**Table 5:** Results of multivariate analysis—independent risk factors for obesity/overweight with various international criteria. CDC—Centers for Disease Control and Prevention, IAP—Indian Academy of Pediatrics, IOTF—International Obesity Task Force criteria, NS—Not Statistically Significant (*p* < 0.05), OR 95% CI—Odds Ratio with 95% Confidence Interval, WHO—World Health Organization.

Risk Factor	IAP Criteria (OR, 95% CI)	CDC Criteria (OR, 95% CI)	WHO Criteria (OR, 95% CI)	IOTF (OR, 95% CI)
Eating meals prepared outside the home, 1–2 times per week	11.92, 2.36 to 60.32	26.49, 3.38 to 207.44	17.69, 3.28 to 95.36	30.19, 3.79 to 240.49
Consumption of sugar-sweetened beverages, 5–7 times per week	9.58, 1.17 to 78.73	12.95, 1.12 to 150.27	NS	7.55, 1.13 to 50.18
Fried food consumption	NS	NS	NS	NS
Physical activity/exercise, 5 or more times per week	0.08, 0.009 to 0.71	0.02, 0.001 to 0.32	0.09, 0.01 to 0.76	0.02, 0.001 to 0.39
Sleep duration, 11 h or more	0.02, 0.002 to 0.36	0.03, 0.002 to 0.47	0.04, 0.003 to 0.46	0.04, 0.003 to 0.61

## Data Availability

The original contributions presented in this study are included in the article and Supplementary Material. Further inquiries can be directed to the corresponding author.

## Supplementary Materials

The following supporting information can be downloaded at: https://www.mdpi.com/article/10.3390/ijerph22111697/s1, File S1: Survey—Prevalence of Risk Factors.

## Abbreviations

The following abbreviations are used in this manuscript:

LMIC	Low- and middle-income country
NAFLD	Nonalcoholic fatty liver disease
BMI	Body Mass Index
IAP	Indian Academy of Pediatrics
WHO	World Health Organization
CDC	Centers for Disease Control and Prevention, United States
IOTF	International Obesity Task Force
OSA	Obstructive sleep apnea
SCFE	Slipped capital femoral epiphysis
PCOS	Polycystic ovarian syndrome
IQR	Interquartile Range
OR	Odds Ratio
CI	Confidence Interval
HFSS	High in fat, sugar, and salt
NS	Not statistically significant
