# Supplementary material for: Risk Factors and Complications of Childhood Obesity and Overweight in an Urban Setting of a Lower Middle-Income Country
Source: Int J Environ Res Public Health. 2025 Nov 10;22(11):1697. doi: 10.3390/ijerph22111697 (PMC12652059; doi:10.3390/ijerph22111697)
Supplement: Supplementary file 1 [file ijerph-22-01697-s001.zip › ijerph-3931505-supplementary.pdf]

SUPPLEMENTARY APPENDIX

SURVEY- PREVALENCE OF RISK FACTORS

1. DIET:
   a. Is your child-
      i. Vegetarian
      ii. Non-vegetarian
   b. How often does your child eat meals outside the house in a week (NOT including school lunches). This includes all meals cooked and purchased outside the home, including meals at restaurants, and meals purchased outside but consumed at home -
      i. Never/ very rarely (No more than once a month)
      ii. 1-2 times
      iii. 3-4 times
      iv. 5 or more times
   c. How many sugar-sweetened drinks (Coca-cola, Pepsi, Fanta, Sprite or any other drink that is sweet and purchased in a store) does your child drink in a week?
      i. Never/ very rarely
      ii. 1-4 drinks
      iii. 5-7 drinks
      iv. 8 or more drinks
   d. How often does your child eat dishes fried in oil every week?
      i. Never/ very rarely
      ii. 1-2 times
      iii. 3-4 times
      iv. 5 or more times
   e. How often does your child eat rice as a major part of their meal?
      i. Never/ very rarely

ii. 1-2 times a week

iii. 3-4 times a week

iv. 5 or more times a week

v. Once per day every day

vi. Twice or more often per day, every day

f. How many servings of vegetables that are NOT fried in oil does your child eat every week?

i. Never/ very rarely

ii. 1-2 times a week

iii. 3-4 times a week

iv. 5 or more times a week

v. Once per day every day

vi. Twice or more often per day every day

g. How many servings of fruits (not juice) does your child eat every week?

i. Never/ very rarely

ii. 1-2 times a week

iii. 3-4 times a week

iv. 5 or more times a week

v. Once per day every day

vi. Twice or more often per day every day

2. EXERCISE: How many times per week does your child play or exercise enough to make him/her breathe hard for 20 or more minutes?

i. Never/ very rarely

ii. 1-2 times

iii. 3-4 times

iv. 5 or more times

3. SCREEN TIME: About how many hours does your child sit and watch/ play each of the following on an average school day?
    a. Television
        i. None
        ii. Less than 1 hour
        iii. 1-2 hours
        iv. 3-4 hours
        v. 5 or more hrs
    b. Computer, other than video games
        i. None
        ii. Less than 1 hour
        iii. 1-2 hours
        iv. 3-4 hours
        v. 5 or more hrs
    c. Mobile Phone or tablet
        i. None
        ii. Less than 1 hour
        iii. 1-2 hours
        iv. 3-4 hours
        v. 5 or more hrs
    d. Video Games
        i. None
        ii. Less than 1 hour
        iii. 1-2 hours
        iv. 3-4 hours
        v. 5 or more hrs
4. SLEEP:

a. What time is bedtime for your child?

    i. Earlier than 7:00pm
    ii. Close to 7:00pm
    iii. Close to 8:00pm
    iv. Close to 9:00pm
    v. Close to 10:00pm
    vi. Close to 11:00pm
    vii. After 11:00pm

b. Approximately how many hours of sleep does your child get every night?

    i. <4
    ii. 4-6
    iii. 6-8
    iv. 8-10
    v. >11
